# A bioelectric model of carcinogenesis, including propagation of cell membrane depolarization and reversal therapies

**DOI:** 10.1038/s41598-021-92951-0

**Published:** 2021-06-30

**Authors:** Joao Carvalho

**Affiliations:** Department of Physics, CFisUC, University of Coimbra, Coimbra, Portugal

**Keywords:** Cancer, Computational biophysics

## Abstract

As the main theory of carcinogenesis, the Somatic Mutation Theory, increasingly presents difficulties to explain some experimental observations, different theories are being proposed. A major alternative approach is the Tissue Organization Field Theory, which explains cancer origin as a tissue regulation disease instead of having a mainly cellular origin. This work fits in the latter hypothesis, proposing the bioelectric field, in particular the cell membrane polarization state, and ionic exchange through ion channels and gap junctions, as an important mechanism of cell communication and tissue organization and regulation. Taking into account recent experimental results and proposed bioelectric models, a computational model of cancer initiation was developed, including the propagation of a cell depolarization wave in the tissue under consideration. Cell depolarization leads to a change in its state, with the activation and deactivation of several regulation pathways, increasing cell proliferation and motility, changing its epigenetic state to a more stem cell-like behavior without the requirement of genomic mutation. The intercellular communication via gap junctions leads, in certain circumstances, to a bioelectric state propagation to neighbor cells, in a chain-like reaction, till an electric discontinuity is reached. However, this is a reversible process, and it was shown experimentally that, by implementing a therapy targeted on cell ion exchange channels, it is possible to reverse the state and repolarize cells. This mechanism can be an important alternative way in cancer prevention, diagnosis and therapy, and new experiments are proposed to test the presented hypothesis.

## Introduction

The current standard theory to explain tumorigenesis is the Somatic Mutation Theory (SMT)^1,2^, which proposes that the origin of cancer can be interpreted by an accumulation of genetic mutations, in particular on tumor suppressor genes and oncogenes, that are passed to their cell descendants. Tumor development is then a multistep process, where successive mutations produce advantageous biological capabilities^3^. This is the widely accepted theory of cancer initiation and it can explain many cancer features, from hereditary cancers to the late onset on life of most of them, and the success of some therapies targeting mutant genes^1^. However, there are many experimental results that contradict SMT, in particular the existence of non-genotoxic carcinogens, like chloroform and p-dichlorobenzene^4^, inducing cancer without DNA alterations, and no mutations are detected in some tumors^5^. Also, changes in the DNA methylation pattern, not in its sequence (only the rate of proteins production, not their composition), are found in some cancerous lesions^1^, and it is possible to induce carcinogenesis without mutagenesis by the introduction of foreign materials into body tissues^6^.

The bioelectric properties of non neural cells have lately attracted considerable attention, with surprising results being reported. These include the regulation of individual cell behavior and organ-level patterns by membrane electric potential and ion fluxes^7,8^, and also the involvement of the bioelectric state in tissue regeneration^9^, heart and muscle patterning^10^ or left-right arrangement^11^. In cancer research there were also several recent discoveries and experiments that show the importance of cells bioelectric state, in particular the cell membrane electric potential and the ion channels conductivity, in cancer initiation and therapy^12^. A relevant work^13^ shows, using the Xenopus laevis model, that gap junctions are important in long-range regulation of tumor formation. In^14^ the authors report on relationships between ion channel dysfunction and cancer hallmarks.

Another example of the influence of the tissue bioelectric properties on cancer, is the effect of tissue denervation, which was shown that can contain tumor growth^15^. It was reported that the progress of precancerous lesions can be delayed, as well as tumor growth rate and metastasis probability reduced, by nerve ablation^16^. It was also demonstrated the importance of an adequate level of bioelectric coupling as suppressor of tumor progress^17^, with the level of connexins expression (related with cells electrical connection via gap junctions) showing a negative correlation with tumor grade^18^. It should be stressed that SMT and TOFT are not mutually exclusive, on the contrary, they are complementary and can both contribute to explain the different circumstances and factors involved in a hugely complex event that is carcinogenesis.

In a recent publication by McNamara et al.^19^, it was demonstrated that, in certain circumstances, tissues that look uniform, can experience a spontaneous bioelectrical spatial symmetry breaking by optogenetic stimulation. This initiates a change of the tissue cells’ bioelectric polarization state, a depolarization or polarization wave which propagates across the domain. The domain wall spreading is qualitatively reproduced in the model presented in the present work.

After this introduction, the next section will advance, in a more detailed way, the relationship between bioelectric bistable states, cells behavior and carcinogenesis. This is followed by a description of the developed bioelectric model, and then by the results obtained in different tests, both with the two and the three-dimensional versions, in diverse conditions. Finally, these results are discussed in the context of carcinogenesis and potential cancer therapies, and some conclusions are extracted, including the proposal of a few possible experimental tests.

## Bioelectric bistability and carcinogenesis

During development of an organism the non-nervous cells’ bioelectric properties evolve considerably, with the electric potential difference across the membrane ($V_m$) changing in space and time^20^. The depolarized state, with $V_m\simeq -10$ to $\simeq -30$ mV, corresponds to more undifferentiated, proliferative and stem-like cells, while the differentiated cells, with a more quiescent behavior, are polarized, with $V_m$ from $\simeq -50$ to $\simeq -90$ mV^21^. It was also found that adult stem cells, proliferative cells and tumor cells are depolarized, and this result motivates the proposal of cells depolarization being at the origin of a tissue tumoral transition^22^, and of the special properties of cancer cells, like uncontrolled proliferation, increased motility and invasion capability. The switch of cell polarity introduces many changes on the genetic expression; for instance, genes like Frizzled can be regulated by membrane voltage gradients^12^. This leads to an under or overexpression of particular genes, not due to any genetic mutation but to a change in membrane polarity^23^. The excessive cell proliferation in the absence of adequate control mechanisms, leads to environmental stress factors (like hypoxia and decreased pH) that can result in genetic instability^24^. The numerous and diverse mutation patterns found in tumors can then be considered as a consequence of carcinogenesis, and all the ensuing chain of events, and not its cause^25^.

Cell depolarization can be caused by some carcinogenic events^26^, being it chemical, ionizing radiation, or any other important perturbation on tissue homeostasis, like the introduction of foreign materials^27^. One example is the radiogenic activation of ${\rm Ca}^{2+}$-activated ${\rm K}^+$ channels and ${\rm Ca}^{2+}$-permeable cation channels, which change the bioelectric cell state and contributes to cell death, but also to DNA repair or lowering the oxidative stress injury^28^. Also ionizing radiation has been shown to activate ${\rm K}^+$ channels^29^. However, this is a tissue wide and not a single cell event, as more than just a few cells are affected. The electric communication between cells, in particular via gap junctions^30^, that allow for ion exchange between neighbor cells, drive the dilution and normalization of cells’ polarization level (a community effect)^31^. So, in normal conditions, in the current proposal, the system is highly resilient and if the perturbation don’t reach a threshold percentage of the cells involved or if it is not concentrated in a small tissue region, the system will return to the default (polarized) state. Otherwise a depolarization wave can develop, reaching most or all of the cells in the electrically connected domain.

The present hypothesis has many contact points with the Tissue Organization Field Theory (TOFT)^32,33^, in the sense that it considers cancer a disease of tissue organization, and a tumor can originate from a perturbation on the tissue environment, not of a single or reduced number of cells, leading to a transition from normal to pluripotent like cells^34^. The bioelectrical communication is then a mechanism for tissue cells homeostasis but also, in case of a major perturbation, a process driving a global cells’ state change. This phenomena is also present during organism development, where spatial and temporal variations in the distribution of membrane electrical potentials are responsible for patterns’ formation, and are involved in such fundamental processes as eye formation, limb regeneration or anatomical axes definition^35^. But a stress (carcinogenic) event can overwhelm tissue control feedback systems and cells have their epigenetic program modified, due to the strong bioelectric coupling with neighbor cells via gap junctions and, eventually, also by intercellular electric fields. The uncontrolled cell number expansion and invasion results in a hostile microenvironment, including hypoxia, nutrient depletion and low pH, which induces mutagenesis, DNA damage and impairment of DNA repair^24,36^.

In the proposed mechanism, hereditary cancers can be explained by transmitted variability, which drive cells to exhibit different electrical properties, in particular in the number, conductivity and efficiency of ion channels, ion pumps and gap junctions. Therefore, particular tissues can be more or less prone to depolarization and sensible to induction of a tumorigenic behavior. It can also explains why cancer is more probable in old age^37^, with the reduced expression of bioelectric state related genes and thus a diminished bioelectric resilience, making the depolarization transition more probable. The cellular electric microenvironment is also perturbed with the increase in number and volume of adipocytes, changing tissues electrical conductivity, permittivity and capacity^38^, and leading to changes in cancer incidence in overweight and obese patients.

A positive outcome of the present hypothesis is that tissue depolarization can be a reversible process, and then tumor development can be controlled, or even reversed, by therapies targeting the cells bioelectric properties. This can be done, as shown in^39^, by stimulation of the potassium/sodium hyperpolarization-activated cyclic nucleotide-gated ion channel 2 (HCN2) to hyperpolarize and/or depolarize different tissue regions and return to normal gene expression in brain teratogenesis^7^. Small molecule drugs can also be used to change the properties of ion channels and change the membrane electric potential^40^, and be used to repair them in case of abnormal activity.

## Methods: computational model

**Figure 1:**
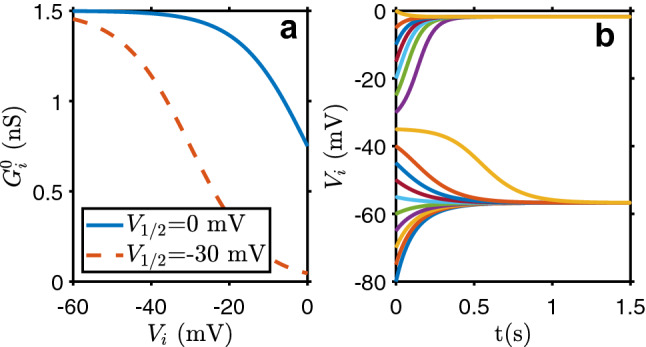
**(a)** Single cell gap junction ionic conductivity as a function of the cell membrane electric potential, as given by Eq. (4), with the parameters used in this work ($V_{1/2}=0$ mV) and the ones used in^31^ ($V_{1/2}=-30$ mV). **(b)** Evolution in time of an isolated cell membrane electric potential for different initial membrane electric potential values. There are two stable points, at $\simeq -2$ mV and $\simeq -57$ mV, with the separation taking place at $\simeq -35$ mV

A cellular automata is implemented to describe a generic tissue cellular organization and its dynamics. The model represents a single layer tissue, in the 2 dimensional version, or a volumetric tissue, with the three dimensional description. The electrical properties of cells and of their interactions closely follow the work developed by Cervera et al., described in detail in^31,41,42^. The simulation of cell ion exchange with other cells is reduced to a generic gap junction, and with the extracellular environment to general depolarization and polarization ion channels. Only the movement of positive ions are considered, and cell *i* electrical potential (relative to the exterior), $V_i$, changes in time according with the equation

1 $$\begin{aligned} C_i \frac{dV_i}{dt} = -I_{\text {dep}} -I_{\text {pol}} + \sum _j^{\text {neigh.}} G_{ij}\left( V_j - V_i\right) \end{aligned}$$

where $C_i$ is the cell membrane capacity, $I_{\text {dep}}$ is the depolarization current (through depolarization ion channels), $I_{\text {pol}}$ the polarization current (by polarization ion channels) and $G_{ij}$ is the ionic conductivity between adjacent cells (through gap junctions; the sum is run over the neighbor cells ). All three terms on the right hand side of the equation depend on the cell electric potential as follows,

2 $$\begin{aligned} I_{\text{ dep }}= & {} \frac{G^0_{\text{ dep }} \left( V_i - E_{\text {dep}}\right) }{1+\exp [-z\left( V_i + V_T\right) /V_T]} \qquad , \qquad I_{\text{ pol }} = \frac{G^0_{\text{ pol }} \left( V_i - E_{\text {pol}}\right) }{1+\exp [z\left( V_i + V_T\right) /V_T]} \end{aligned}$$

3 $$\begin{aligned} G_{ij}= & {} \frac{2G_{ij}^0}{1+\cosh [\left( V_i - V_j\right) /V_0]} \end{aligned}$$

with

4 $$\begin{aligned} G_{ij}^0 = \frac{G_{i}^0 G_{j}^0}{G_{i}^0 + G_{j}^0} \qquad , \qquad G_{i}^0 = \frac{G_{\text{ max }}^0}{1+\exp [z\left( V_i - V_{1/2}\right) /V_T]} \end{aligned}$$

where $E_{\text{ dep }}$ ($E_{\text{ pol }}$) is the depolarization (polarization) equilibrium potential, $V_T$ the threshold potential, *z* the channel gating charge, $G^0_{\text {dep}}$ ($G^0_{\text {pol}}$) the conductance of the depolarization (polarization) ion channels, $V_{1/2}$ the potential for which $G_{i}^0$ decreases by a factor 2 (represented in Fig. 1a), and $V_0$ controls the width of the gap junction conductivity function^31^. The standard values of the model parameters, based on experimental results, are presented in^31^ and given in table 1. With these values the cells electric potential tend to a value of $\simeq -57$ mV in a polarized domain and to $\simeq -2$ mV in a depolarized one, with the separation between the two stable points at $\simeq -35$ mV (see Fig. 1b). $G_{ij}$ describes the gap junctions ionic conductivity between neighbor cells, including the serial association of cells *i* and *j* conductivities ($G_{i}^0$ and $G_{j}^0$). The different values of $G^0_{\text{ dep }}$ and $G^0_{\text{ pol }}$, and the parameter $V_{1/2}$, introduce different conductivities for polarization and depolarization channels. Therefore the system presents a dynamic ion flow behavior driving changes on the membrane electrical potential, being then possible to depolarize and repolarize a tissue.

**Table 1:** Standard cell electrical parameter values used on the model. $\sigma =3$ is applied as the standard deviation on normally distributed values around the mean (stochastic model)

Parameters	Mean value	Standard deviation
$C_i$	100 pF	$0.02 \sigma C_i$ pF
$G_{\text{ ref }}^0$	1 nS	0
$G^0_{\text{ pol }}$	$G^0_{\text{ ref }}$	$0.02\sigma G_{\text{ pol }}^0$ nS
$G^0_{\text{ dep }}$	$2.0 G^0_{\text{ ref }}$	$0.02\sigma G_{\text{ dep }}^0$ nS
$G^0_{\text{ max }}$	$1.5 G^0_{\text{ ref }}$	$0.02\sigma G_{\text{ max }}^0$ nS
*z*	3	0.1
$E_{\text{ dep }}$	0 mV	$\sigma$ mV
$E_{\text{ pol }}$	− 60 mV	$\sigma$ mV
$V_T$	26 mV	$\sigma$ mV
$V_0$	24 mV	0
$V_{1/2}$	0 mV	$\sigma$ mV

But cells are not exactly identical, and, in this model, cell diversity is introduced with the main cell parameters values following a normal distribution, with the mean given in the second column of Table 1 and its standard deviation in the third column of the same table.

A standard run of the cellular automata, for a quiescent tissue, is initiated with all cells polarized, with an average membrane electrical potential $V_m$ centered at $E_{\text{ pol }}=-60$ mV, and distributed according to a gaussian function with $\sigma =3$ mV. The cells being simulated are generic differentiated tissue cells. The domain contains $100\times 100$ ($50\times 50\times 50$) cells in the 2D (3D) version. In the depolarization tests is introduced a patch of depolarized cells, with diverse shapes and locations, with $E_{\text{ dep }}=0$ mV, and the evolution in time of cells membrane electric potential follows Eq. (1) (a Moore neighborhood is used on the sum, with the 8 (26) closest cells being considered the vicinity in 2D (3D); a Neumann neighborhood was also tried, with qualitatively similar results).

Even if based on it, with many common features and parameters, important differences exist between the present model and the Cervera et al. one^31^. Besides being a simpler cellular automata, without taking into account the cell’s shape, it is a stochastic one, introducing variations on cells’ parameters to simulate diversity. A 3D version is also developed and run. The parameter $V_{1/2}=0$ mV is very different from the value − 30 mV employed in the paper. This change reproduces better the difference between polarized and depolarized cells gap junctions conductivity, as reported in^43^. There, the maximum permeability (the maximum ion flux between any two cells), varies between 0.45 for a pluripotent cell and 0.85 for a differentiated cell, consistent with $V_{1/2}=0$ mV. The conductivity behavior for the two values of $V_{1/2}$ considered is shown in Fig. 1a. Also $G^0_{\text{ dep }}=1.5\times G^0_{\text{ pol }}$ in^31^, being $G^0_{\text{ dep }}=2.0\times G^0_{\text{ pol }}$ in the present work, due to its importance to reproduce depolarization waves, as reported in^19^.

It is possible to produce time-stable spatial-varying patterns using cyclic cellular automata rules, involving a small number of stable states, a set of interacting neighbors and some defined rule for state transition (a function of the neighbors’ state)^44^. This is not the subject of the current work but can be implemented in a future study. Electrically segregated states allow for the existence of cells’ compartments in different polarization states, as niches of stem cells.

## Model results

**Figure 2:**
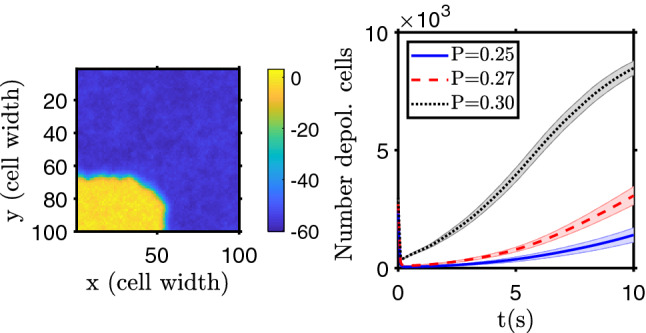
Tissue depolarization for randomly distributed depolarized cells on a polarized domain. Left: example of the two-dimensional domain polarization state after 10 seconds (yellow corresponds to depolarized cells and blue to polarized ones; the color bar shows the membrane electrical potential in mV). Figure S3 shows the spatial distribution of the depolarized cells at different time points, and Supplementary Movie 3 shows an animation of the system evolution in space and time. Right: evolution of the number of depolarized cells for different percentages of depolarized cells randomly initially distributed on the domain (25%, 27% and 30% of the total number of cells depolarized). The initial decrease on the number of depolarized cells (see Fig. S2 for the same plot in semi-logarithmic scale for a more detailed display of the first time steps) is due to a community effect, where depolarized cells with a high number of polarized neighbors will polarize fast. The bands show the standard deviation of the mean of $n=20$ simulation runs.

The square (cubic) domain in 2D (3D) represents an electrically connected tissue, separated from the vicinity by electrical walls comprised of non electrically connected cells, basal membranes and/or extracellular matrix. When the tissue domain is initiated, with all cells in a depolarized or a polarized state, it keeps the same of the two possible bistable states, being resilient to a limited number of cells’ polarization state flips. Starting with a polarized domain, like in a quiescent tissue, if the number of (randomly chosen) cells that become depolarized exceeds a certain threshold, about 25% for 2D and about 39% for 3D, then there is a chain reaction, due to gap junction communication, and a depolarization wave will run over the domain and all, or almost all, the cells become depolarized in the first 10 s after perturbation. An example of the final cells’ polarization distribution and the number of depolarized cells as function of time are shown in Fig. 2 (for the 3D model, results are shown as supplementary material in Fig. S1; the number of depolarized cells as a function of time, with time in logarithmic scale, is shown in Supplementary Fig. S2). The evolution of depolarization in time and space is also shown in the Supplementary Movie 3.

**Figure 3:**
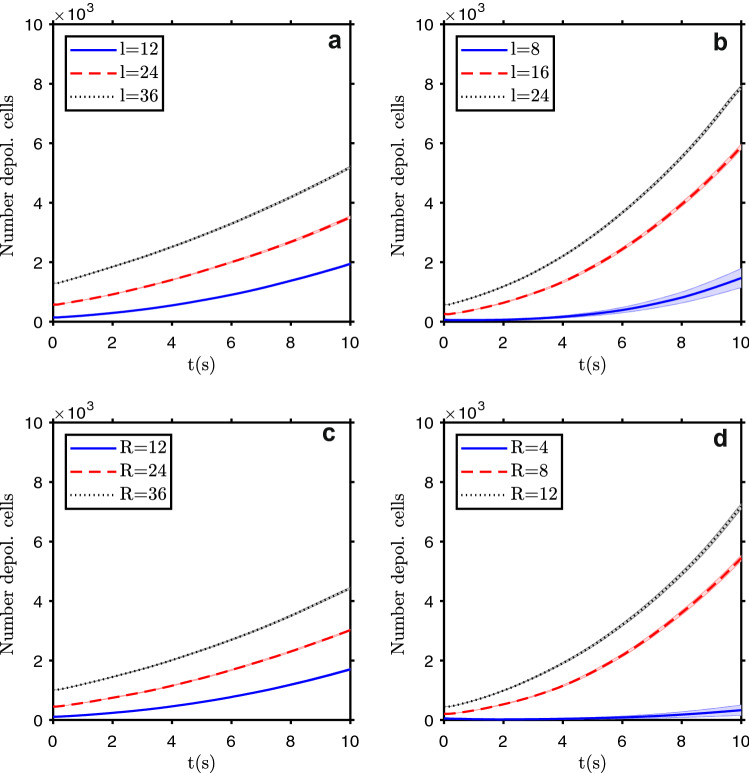
Tissue depolarization after the introduction of a patch of depolarized cells on a polarized tissue. Top row: evolution of the number of depolarized cells for different sizes of a square patch, introduced on the top left corner of the domain, with a width of 12, 24, and 36 cells **(a)**, and on the domain center, with a width of 8, 16, and 24 cells **(b)**. Bottom row: evolution of the number of depolarized cells for different sizes of a circular patch, with a radius of 12, 24, and 36 cells, centered on the top left corner of the domain **(c)**, and placed on the domain center, with a radius of 4, 8 and 12 cells **(d)**. The bands show the standard deviation of the mean of $n=20$ simulation runs.

When the depolarization in concentrated in a local domain, the minimum number of depolarized cells that can initiate the depolarization wave depends on the shape and location of the patch, and also of the dimensionality of the domain. As the cells are similar but not exactly equal, both on their individual properties and their location, the system evolution is different from run to run. Figure 3 shows the evolution of the number of depolarized cells for 10 s, in different conditions, in a 2D domain. To start a depolarization wave, the minimum number of cells on the depolarized patch is about $6\times 6$ for a square patch in a corner of the domain, $\simeq 8\times 8$ if it is at the center. For a circular patch, shown also in Fig. 3, it has a minimum radius of about $R=6$ if a quarter circle is depolarized, centered at a domain corner, and a radius of about $R=4$ if a full circle is centered on the domain (all dimensions are in units of cell width). For the 3D version, shown as supplementary material in Fig. S4, the equivalent numbers are about $12\times 12\times 12$ for a cubic patch in the corner of the domain, about $24\times 24\times 24$ if it is placed at the center, has a radius of around $R=15$ if an eighth of a sphere is depolarized, centered at a domain corner, and a radius of about $R=15$ if a full sphere is placed at the domain center. These results stress the importance of the vicinity (community) effects, with a higher dimensionality domain, where cells’ contact with a higher number of neighbors, needing a larger number of cells to break the system resilience and start the depolarization wave. This also points to the importance of bioelectricity studies in vivo, where the full electrical conditions can be reproduced to achieve reliable measurements, including the neighborhood effects and the presence of regions with different electrical conductivity, which is not possible to fully replicate in vitro. A recent technique to measure cell polarization uses a voltage reporter dye^35^ to visualize gradients of $V_m$ across tissues. In Supplementary Material are available four examples of simulation animations of the depolarization and polarization wave evolution in different conditions (Supplementary Movie 1 to 4), and in Fig. S3 the system state at different time points.

**Figure 4:**
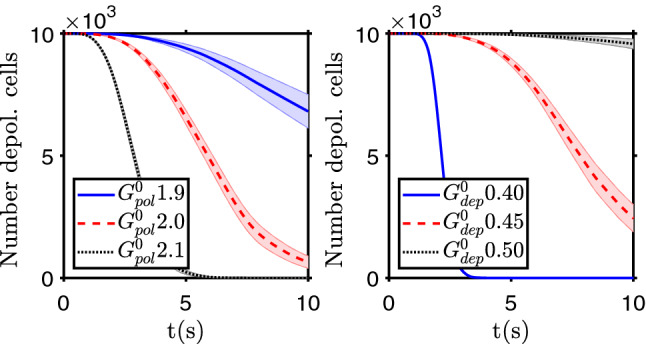
Repolarization therapies. Evolution of the number of depolarized cells for therapies that increase the polarization ion channel conductivity ($G^0_ {\text{ pol }}$, left) or decrease the depolarization ion channel conductivity ($G^0_ {\text{ dep }}$, right). Initially all the cells were depolarized. The bands show the standard deviation of the mean of $n=20$ simulation runs.

After a tissue is depolarized, and cells enter into a more proliferative and motile state, the risk of it evolving to a tumor greatly increases. This altered behavior can also be associated with increased stressful conditions that can induce genomic mutations^24^. But this event can be reversed, if it is possible to change the number or conductivity of ion channels, ion pumps and/or gap junctions. Experimentally it was shown that, in some conditions, this is achievable^12^. In this model, a repolarization therapy is introduced by a change on cells ionic conductivity, for instance an increase by a factor 2 of the cells polarization ion channel conductivity ($G^0_{\text{ pol }}$) or a decrease by a factor $\sim 0.5$ of cells depolarization ion channel conductivity ($G^0_{\text{ dep }}$). In Fig. 4 it is shown an example of the cells’ polarization state after 10 s and also how the number of polarized cells changes with time after the repolarization intervention.

**Figure 5:**
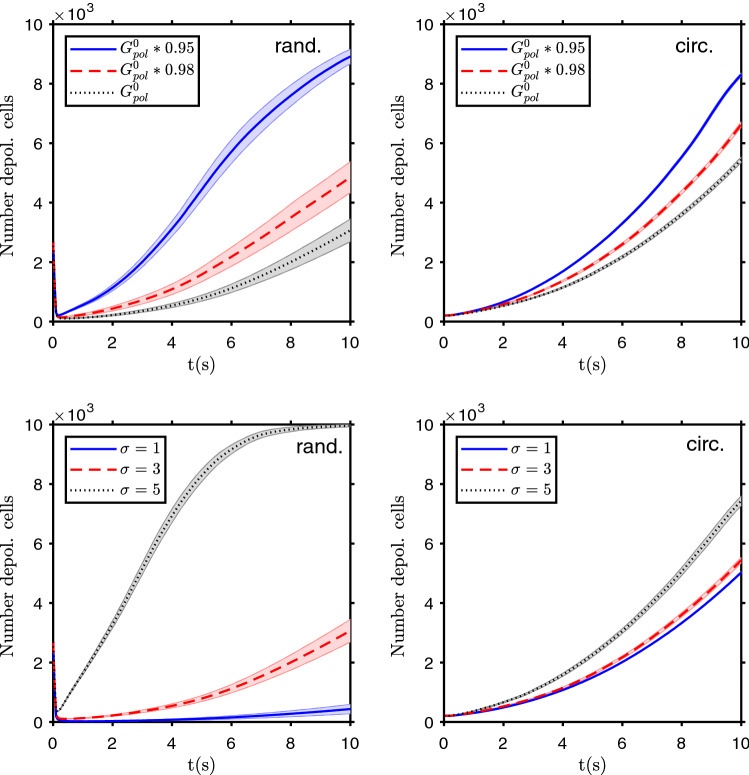
Parameters sensitivity tests. Top row: evolution of the number of depolarized cells for different values of the cells polarization channel conductivity $G^0_ {\text{ pol }}$. Bottom row: evolution of the number of depolarized cells for different values of the standard deviation $\sigma$ of the cells’ bioelectric properties. The results are shown for 27% of depolarized cells randomly distributed on the domain at the start of the simulation (left column), and for an initial circular depolarization patch (with $R=8$ cells’ width) at the center of the domain (right column). There is a saturation at 10 k cells, the total number of cells in the domain. The bands show the standard deviation of the mean of $n=20$ simulation runs.

Due to hereditary variation, or a change with aging, on the number and/or conductivity of ion and gap junction channels, some persons (and/or some particular tissues) can be more susceptible to initiate a depolarization wave, and then to carcinogenesis, due to these bioelectric effects. The change on ion channels expression or performance can also be due to carcinogenic events, like ionizing radiation or chemical agents. These possible consequences are simulated by small changes on cells’ polarization ion channels conductivity, $G^0_ {\text{ pol }}$, due to, for instance, a decrease on the number of these channels. Figure 5 shows the number of depolarized cells as a function of time for depolarization events. It is clear that a decrease of $G^0_ {\text{ pol }}$, even small, makes the depolarization faster and more probable. Also aging, or diverse hereditary genomic or epigenomic expression, can increase the variability on the cells bioelectric properties. This is simulated by a variation of the standard deviation $\sigma$ value given on Table 1, and the results are shown in Fig. 5. It is clear that, as the variability increases, the depolarization is also faster and more probable.

**Figure 6:**
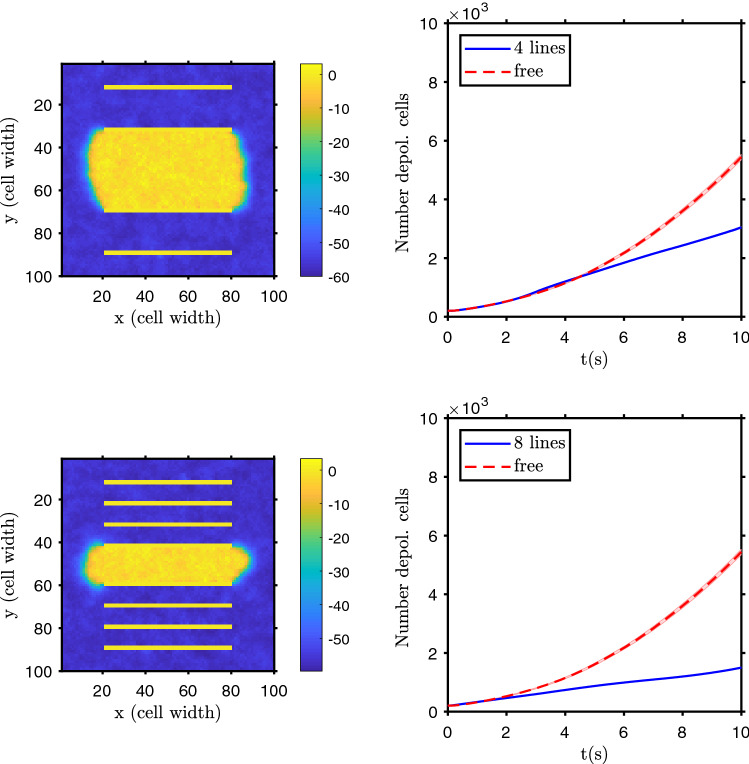
Effect of electrical isolating obstacles. Example of the two-dimensional domain polarization state after 10 seconds (left column, yellow corresponds to depolarized cells and blue to polarized ones) and the evolution of the number of depolarized cells (right column) for a domain with 4 isolating walls (top row, yellow horizontal bands) and with 8 isolating walls (bottom row). Initially all the cells were depolarized and an initial circular depolarized patch, with $R=8$ cells’ width, is placed on the center of the domain. The bands show the standard deviation of the mean of $n=20$ simulation runs.

The introduction of electric walls on the tissue, with regions electrically isolated from others, where cells don’t have as many neighbors to exchange ions through gap junctions, leads, naturally, to a reduction of the community effects. The depolarization wave is slower and the probability of starting one decreases, as shown in Fig. 6.

**Figure 7:**
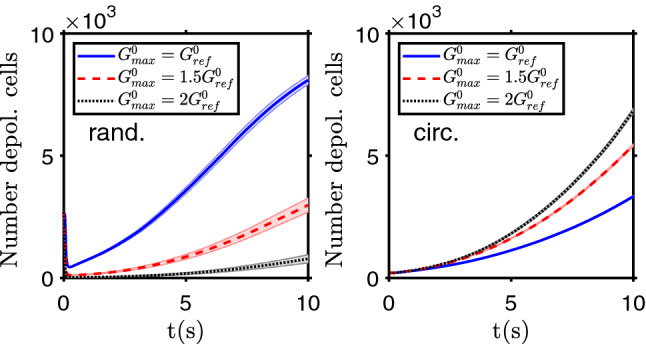
Community effect. Evolution of the number of depolarized cells for different values of the gap junction conductivity parameter $G^0_ {\text{ max }}$. The depolarization starts from a random distribution of depolarized cells, 27% of the total (left), or from a circle of depolarized cells, with $R=8$ cells’ width, at the center of the two dimensional domain (right). The bands show the standard deviation of the mean of $n=50$ simulation runs.

Increasing or decreasing the gap junction conductivity, by a change of the $G^0_ {\text{ max }}$ parameter, leads to apparently counterintuitive results. The gap junctions are important both to keep the tissue bioelectric state, the system homeostasis, by “diluting” the excess charge in one cell with the cells connected with it, and then leaning to keep a similar state among neighbors. But it is also fundamental for the depolarization or polarization wave propagation, with cells changing their state due to the influence of the neighbor cells by ion transfer via gap junctions. This is shown in Fig. 7, where for an increase or a decrease of $G^0_ {\text{ max }}$, the probability and speed of depolarization changes in opposite ways as compared with the standard parameter value $G^0_ {\text{ max }} =1.5 G^0_ {\text{ ref }}$, depending on the initial conditions. For dispersed small clusters of depolarized cells, when they are randomly distributed on the domain and in the presence of more numerous polarized ones, the community effect goes against most of the depolarized cells, with the influence of the neighbors leading to the repolarization of nearly all cells, when there is a high connectivity via gap junctions. So the speed of depolarization decreases with the increase on $G^0_ {\text{ max }}$. In the second case, when there is an initial large cluster of depolarized cells, it prompts the depolarization of the neighbor cells, and this effect is stronger and faster when the ionic conductivity via gap junctions is larger. The first circumstances can be applied to a consequence of aging, which leads to a decrease of gap junctions number and/or activity^45,47^ and then made more probable the depolarization wave (and then carcinogenesis) from random depolarization events. The control of gap junctions conductivity can be a target for cancer prevention therapies and the inhibition of gap junctions improved the chemotherapy response of glioblastoma cells^46^.

In McNamara et al.^19^ it is reported, in supplementary figure 2, the depolarization domain wall velocity, which is of the order of magnitude of 1 mm/s. The present model, considering cells with a width of 10 $\mu {\rm m}$, the velocity is $\sim 0.040$ mm/s, but using the same cell electric capacitance as in the experimental article, $C_i=10$ pF, it is 10 times faster, or 0.4 mm/s, in good agreement with the experimental results.

## Discussion and conclusions

A simple model of bioelectric community effects was enhanced and applied to carcinogenesis, showing bistability, where the depolarized state corresponds to a more proliferative and motile cell (and then a more cancer cell-like state)^48^, and the polarized state to a quiescent behavior. The results point to the importance of the neighborhood in inducing a particular cell state, not just the single cell properties, as determinant of its bioelectric condition. A polarized tissue, with cells in a quiescent state, tend to continue in that condition except if a large enough perturbation changes the homeostatic circumstances (for instance, by a carcinogenic event). In such an experience, the induced depolarized state can propagate to the neighbors in a wave like fashion. The disruption can be a general one, covering a large percentage of cells on the tissue (as due to hereditary conditions or consequence of aging), or a localized one, where the community effects lead to an expansion of the depolarized state. However, a fully depolarized tissue can also return to the polarized condition, if the cells electrical conductivity or the ion pumps activity is changed in the right direction.

Even if the cells’ bioelectric state and the ion exchange with neighbors are not the only relevant factors on carcinogenesis, this is a hypothesis that merit to be considered. Dedicated experiments should be pursued on the association between bioelectricity, in particular the membrane electric potential and its relation with cell regulation pathways, and cancer inception. Being it, as much of the scientific evidence shows, a multifactorial disease, it is fundamental to understand how the cell state can be modified by changes on tissue regulation, in particular on ion channels activity and overall electrical properties.

Also very relevant are the community effects associated with cell electrical communication, leading to both short and long range influence and patterns^42^, and their importance in cancer, as the link between the cells’ bioelectric state and invasiveness^49^ or the role signaling by calcium permeable channels play in tumor progress^50^. The manipulation of the tissue bioelectric state, profiting from the long range impact and propagation of the polarization state, can be an effective and efficient approach for cancer prevention and therapy. Besides the small molecules drugs that can be used on cells’ ion channels and pumps^51^, a long odd would be to use static electric fields and/or electromagnetic fields (EMF) to reverse the cancerous tissue to a more quiescent behavior. Examples of effects of EMF on ion channels are, for instance, the stimulation of voltage-gated calcium channels^52^, the inhibition of cancer cells growth by the promotion of voltage-gated T-type ${\rm Ca}^{2+}$ channels activity^53^, or the cells’ transmembrane potential change by nanosecond pulsed electric fields, which act on specific voltage-sensitive ion channels^54^. Likewise, it can also be tried to reverse the state modifications by changing the electrical microenvironment. Additionally it can be adjusted by changing the tissue electrical properties, by a treatment with drugs or normal tissue components, with, for instance, different electrical conductivity.

The model presented in this work shows not only the importance of the community effects, with much stronger response when the number of neighbors increases, as when one goes from two to three dimensions, or decreases, when electrical segregating elements are introduced. It also shows the importance of cells bioelectric variability, making it easier to create the conditions to start the tissue depolarization, and then for cells to change their state from quiescence to stem-like (proliferative and motile).

There is an increased volume of evidence on the relation between carcinogenesis and the tissue bioelectric state^55^. This effort should be reinforced with additional focused experiments, in particular using in vivo models, the only way to reliably reproduce tissues bioelectric properties^35^. The importance of the confirmation of these mechanisms, even if covering only part of the cancer initiation cases, cannot be overstated; this opens alternative avenues in cancer research, not only in prevention and diagnosis, but mainly in therapy, with the possibility of reversing a tumor to a healthy tissue.

In order to further investigate and develop this hypothesis of a relevant relation between a tissue bioelectric state and carcinogenesis, there are a set of new experiments and measurements that should be envisaged. These include the ones to establish how a carcinogenic event (chemical factors, ionizing radiation, etc.) changes the tissue bioelectric state, and, in particular, the cells ion channels and pumps, gap junctions and electrical conductivity. It is also important a more detailed study of the ion channels properties in non neural cells of different tissues, specially the gap junctions characteristics in distinct in vivo conditions. Likewise relevant is a specific study of the importance of the membrane electric potential on critical regulatory pathways and genetic expression related with the cancer hallmarks^3^, and with genomic instability and DNA repair mechanisms^56^. It is still fundamental to prosecute the research on the capability of small molecules, electric fields and non-ionizing radiation to change the bioelectrical properties of the cells, concerning their use for cancer prevention and therapy^57^. Finally, it is necessary to search for relations between the diverse cell and tissue inputs, being them electrical, chemical or mechanical, and the tissue organization; a theory of organization, regulation and information in organisms is paramount for an educated investigation of carcinogenesis mechanisms^58,59^. To predict the cell response, the bioelectric tissue state must be associated with other information received by the cell, in particular chemical and mechanical signaling. So it is essential to combine the bioelectric state with genetic signaling networks, as, in general, the gene expression prompts the bioelectric state, but this direction of action can, in some cases, be reversed^19^.

## Supplementary Information

Supplementary Information.

Supplementary Video 1.

Supplementary Video 2.

Supplementary Video 3.

Supplementary Video 4.

## Data Availability

The matlab code used in the simulations is available from the author upon request.
