# Supplementary material for: A bioelectric model of carcinogenesis, including propagation of cell membrane depolarization and reversal therapies
Source: Sci Rep. 2021 Jun 30;11:13607. doi: 10.1038/s41598-021-92951-0 (PMC8245601; doi:10.1038/s41598-021-92951-0)
Supplement: Supplementary file 1 — Supplementary Information. [file 41598_2021_92951_MOESM1_ESM.pdf]

A bioelectric model of carcinogenesis, including propagation of cell membrane depolarization and reversal therapies

Joao Carvalho, CFisUC, Department of Physics, University of Coimbra, Portugal

Supplementary Material

Supplementary Movie 1: Animation of the evolution of cells' polarization state in a polarized two dimensional domain, from a square depolarized patch on a domain corner (yellow corresponds to depolarized cells and blue to polarized ones).

Supplementary Movie 2: Animation of the evolution of cells' polarization state in a polarized two dimensional domain, from a circular depolarized patch at the center of the domain (yellow corresponds to depolarized cells and blue to polarized ones).

Supplementary Movie 3: Animation of the evolution of cells' polarization state in a polarized two dimensional domain, from a random distribution of depolarized cells on the domain (yellow corresponds to depolarized cells and blue to polarized ones).

Supplementary Movie 4: Animation of the evolution of cells' polarization state in a depolarized two dimensional domain, for therapies that increase the polarization ion channel conductivity (G^0_{pol}) (yellow corresponds to depolarized cells and blue to polarized ones).

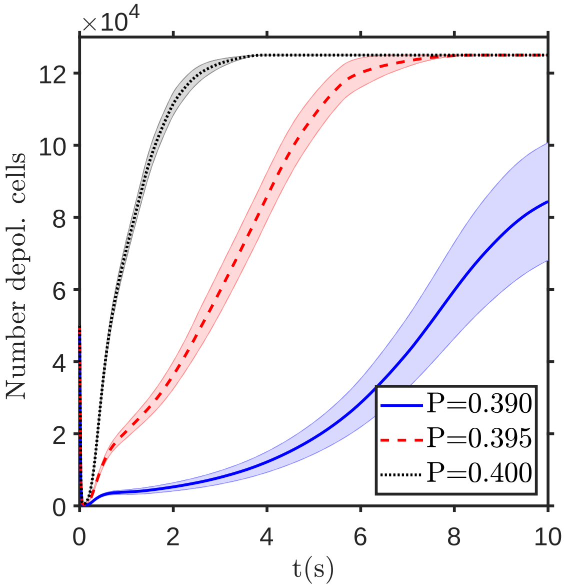

Figure S1: Evolution of the number of depolarized cells for different numbers of depolarized cells randomly distributed in a polarized tissue, in a three dimensional domain. The sharp initial decrease on the number of depolarized cells is due to community effects, when depolarized cells are surrounded by a majority of polarized ones. The number of depolarized cells saturates when all cells on the domain depolarize. The bands show the standard deviation of the mean of $n = 10$ simulation runs.

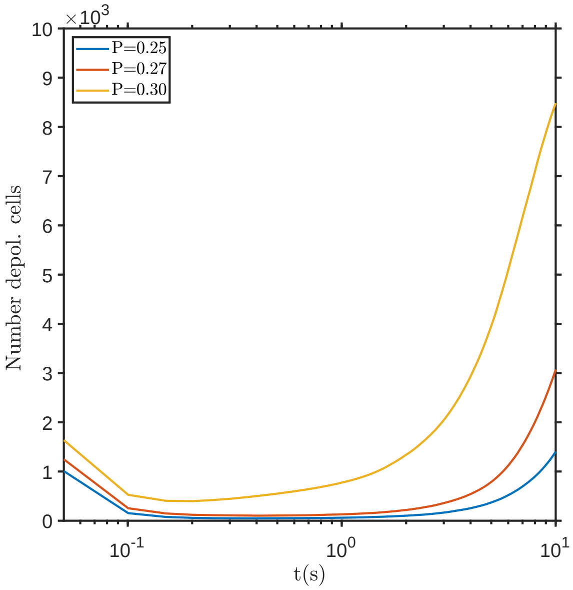

Figure S2: Tissue depolarization for randomly distributed depolarized cells on a polarized domain. Evolution of the number of depolarized cells for different percentages of depolarized cells randomly distributed on the domain (25%, 27% and 30% of the total number of cells depolarized). The initial sharp decrease on the number of depolarized cells is due to a community effect, where depolarized cells with a high number of polarized neighbors will polarize fast. Note that a semi-logarithmic scale is being used to highlight the fast change at the start of simulation.

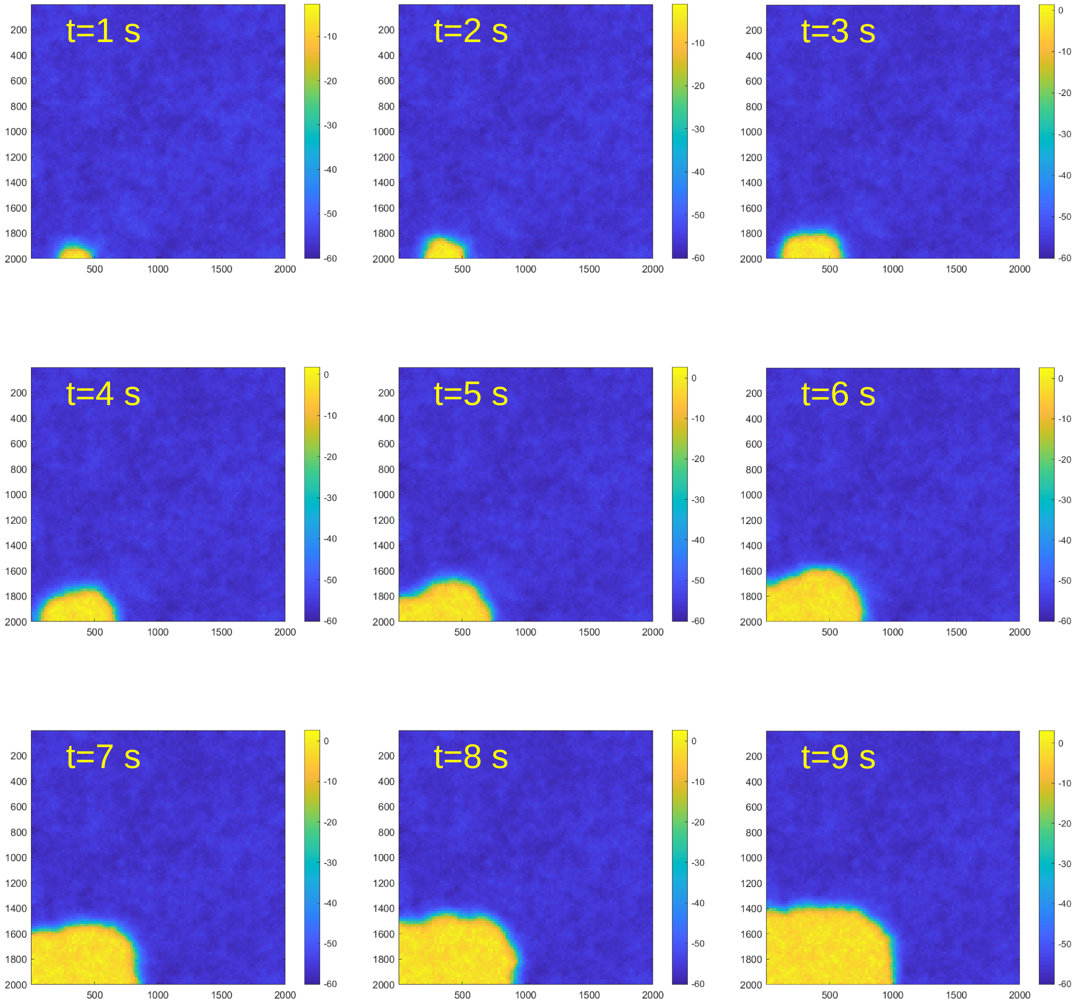

Figure S3: Tissue depolarization for randomly distributed depolarized cells on a polarized domain. Example of the two-dimensional domain polarization state at different time steps (yellow corresponds to depolarized cells and blue to polarized ones; the color bar shows the membrane electrical potential in mV). The initial depolarized region is located in a random position; it is the place where the initial depolarized cells concentration is high enough to overcome the polarization effect from neighbor cells.

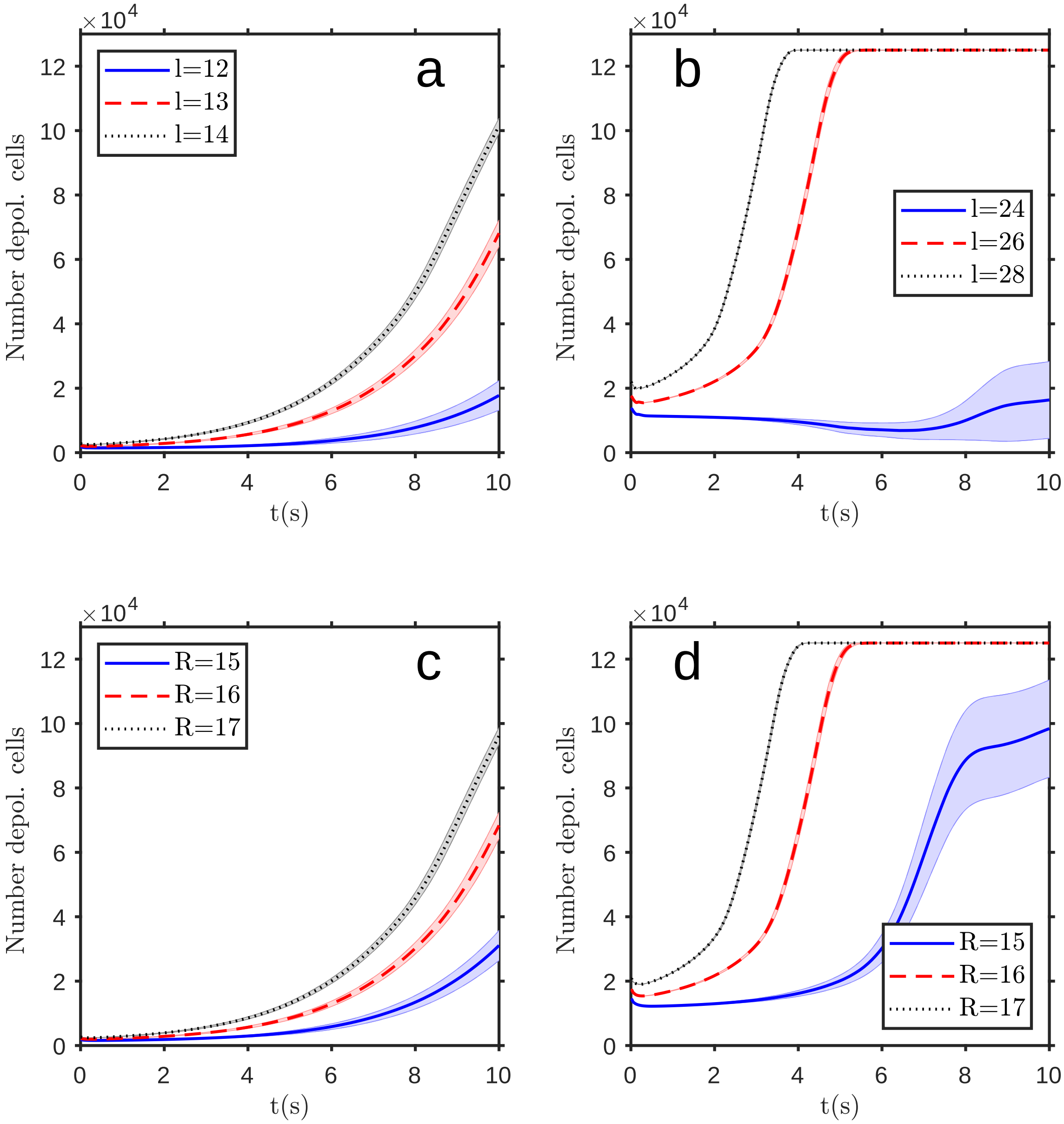

Figure S4: Tissue depolarization after the introduction of a patch of depolarized cells on a polarized tissue. Evolution of the number of depolarized cells for different geometries and sizes, in a three dimensional domain. a) cubic patch on a domain corner; b) cubic patch on a domain center; c) 1/8 of a sphere, centered on a domain corner; d) spherical patch, centered on the domain. The bands show the standard deviation of the mean of $n = 10$ simulation runs.
